# Noncoding Genomics in Gastric Cancer and the Gastric Precancerous Cascade: Pathogenesis and Biomarkers

**DOI:** 10.1155/2015/503762

**Published:** 2015-08-26

**Authors:** Alejandra Sandoval-Bórquez, Kathleen Saavedra, Gonzalo Carrasco-Avino, Benjamin Garcia-Bloj, Jacqueline Fry, Ignacio Wichmann, Alejandro H. Corvalán

**Affiliations:** ^1^Advanced Center for Chronic Diseases (ACCDiS), Pontificia Universidad Católica de Chile, 8330034 Santiago, Chile; ^2^Scientific and Technological Bioresource Nucleus (BIOREN) and Graduate Program in Applied Cell and Molecular Biology, Universidad de La Frontera, 4811230 Temuco, Chile; ^3^UC-Center for Investigational Oncology (CITO), Pontificia Universidad Católica de Chile, 8330034 Santiago, Chile; ^4^Department of Pathology, Universidad de Chile, 8380453 Santiago, Chile; ^5^Department of Pathology, Icahn School of Medicine at Mount Sinai, New York 10029, NY, USA; ^6^Department of Hematology-Oncology, Faculty of Medicine, Pontificia Universidad Católica de Chile, 8330034 Santiago, Chile

## Abstract

Gastric cancer is the fifth most common cancer and the third leading cause of cancer-related death, whose patterns vary among geographical regions and ethnicities. It is a multifactorial disease, and its development depends on infection by *Helicobacter pylori* (*H. pylori*) and Epstein-Barr virus (EBV), host genetic factors, and environmental factors. The heterogeneity of the disease has begun to be unraveled by a comprehensive mutational evaluation of primary tumors. The low-abundance of mutations suggests that other mechanisms participate in the evolution of the disease, such as those found through analyses of noncoding genomics. Noncoding genomics includes single nucleotide polymorphisms (SNPs), regulation of gene expression through DNA methylation of promoter sites, miRNAs, other noncoding RNAs in regulatory regions, and other topics. These processes and molecules ultimately control gene expression. Potential biomarkers are appearing from analyses of noncoding genomics. This review focuses on noncoding genomics and potential biomarkers in the context of gastric cancer and the gastric precancerous cascade.

## 1. Introduction

Gastric cancer is the fifth most common cancer and the third leading cause of cancer-related death in both sexes worldwide, with 723,000 deaths in 2012 [1]. Global patterns of incidence and mortality vary among geographical regions as well as with ethnicities [2–5] contributing to the heterogeneity of gastric cancer and precancerous lesions. This heterogeneity is associated with the multifactorial origin of the disease, which includes infectious agents, host genetic factors, and environmental factors. Infectious agents* Helicobacter pylori* (*H. pylori*) and Epstein-Barr virus (EBV) [6, 7] are considered key players in the carcinogenic process. Chronic infection by* H. pylori* is the strongest known risk factor and is related to the presence of bacterial virulence factors including SabA, BabA, CagA, and VacA. Cag(+) and VacA with allelic variant s1-m1 strains are associated with increased disease severity [8]. In addition,* H. pylori* infection disrupts cell polarity resulting in translocation of beta-catenin and p120 to the nucleus, altering proliferation and apoptosis [8]. Based on monoclonal expression of EBV-encoded small RNA type-1 and elevation of serum antibodies against viral capsid antigen [9], chronic EBV infection has been found in 10%–20% of gastric cancers [10, 11]. Distinctive EBV strains have been identified in EBV-associated gastric cancer [12–14] and EBV tumors have been related to a lower TNM stage and survival advantage [15, 16]. Polymorphisms in IL-1*β*, TNF-*γ*, and IL-10 are host-dependent genetic factors which also contribute to the multifactorial origin of gastric cancer [17]. Environmental factors, such as smoking and alcohol consumption [18], also play an important role in gastric carcinogenesis. In spite of these long-term recognized associations, the molecular bases of gastric cancer and precancerous lesions are just beginning to be unraveled [10]. These emerging data show that mutations in coding genes are not the only relevant factors in carcinogenesis, but also alterations in the noncoding regions of the genome [10, 19]. Noncoding genomics, which ultimately controls the transcriptional machinery, includes single nucleotide polymorphisms (SNPs), DNA methylation, miRNAs in regulatory regions, and other noncoding RNAs, among other topics. Histone modification is one of these other fields within noncoding genomics during malignant transformation, which is beyond the scope of this review and we refer the reader to other excellent and extensive recent reviews in this area [20, 21]. Based on an emerging body of evidence, noncoding genomics may be used for the discovery of novel and specific biomarkers, urgently needed to improve screening results and reduce gastric cancer mortality. This review focuses on the role of the noncoding genome in the pathogenesis of gastric cancer and the gastric precancerous cascade.

## 2. The Framework of Gastric Cancer and the Precancerous Cascade

**Figure 1:**
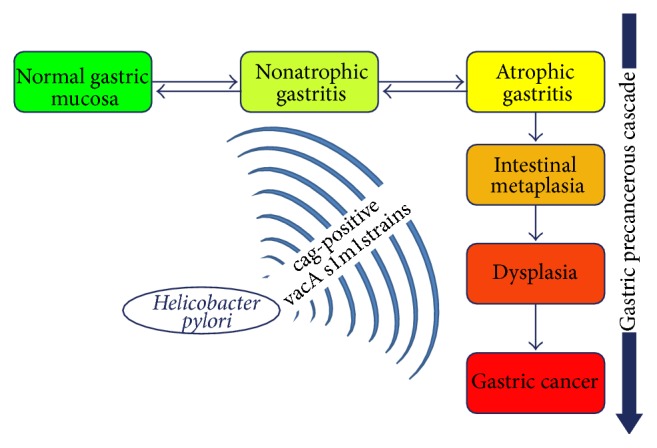
Sequence of lesions produced by* Helicobacter pylori* infection (cag-positive vacA s1m1 is the most aggressive strain). The precancerous cascade starts with atrophic gastritis, which progresses to intestinal metaplasia, dysplasia, and, finally, gastric cancer.

Gastric cancer can be classified as papillary, tubular, mucinous (colloid), and poorly cohesive carcinomas by the World Health Organization [22], as well as intestinal- and diffuse-type by Lauren's classification [23]. This latter classification has been particularly useful for epidemiological studies [24]. The diffuse-type is relatively more frequent in low-risk populations and, when associated with germline mutations of the CDH-1 gene, constitutes the Hereditary Diffuse Gastric Cancer Syndrome (HDGCS) [25]. Diffuse-type gastric cancer has also been associated with Early-Onset Gastric Cancer, a syndrome which occurs before 45 years of age and is linked to CDH-1 gene mutation or BRCA-1 methylation [26–28]. Intestinal-type gastric cancer predominates in high-risk populations and is preceded by a prolonged precancerous process [29]. The precancerous process is very complex, part of which results in a transformation of the normal mucosa into superficial gastritis (SG), chronic nonatrophic gastritis (NAG), chronic atrophic gastritis (AG), intestinal metaplasia (IM), and dysplasia. This process constitutes the basis for the human model of gastric carcinogenesis, known as the gastric precancerous cascade [29] (Figure 1).* H. pylori* infection is the first step of the cascade, producing SG. The following sequential steps include NAG, multifocal AG, IM of the complete type, IM of the incomplete type, low-grade dysplasia, high-grade dysplasia (high-grade noninvasive neoplasia), and invasive adenocarcinoma [30]. The progression through the cascade is a dynamic process of forwards and backwards between less and more advanced lesions, reaching short periods of stability between these episodes of progression and regression. The whole process may last decades. The initial lesion may regress by eradicating* H. pylori* [31]. Several studies have shown that the progression of the precancerous cascade is more likely with a baseline diagnosis of IM or a higher degree lesion (i.e., dysplasia). Therefore, the presence of IM seems to be the “point of no return” in the process, regardless of* H. pylori* eradication [32]. IM is subsequently divided into complete (type I or small intestine) and incomplete (type II or colonic) subtypes depending on their mucin profiling. Incomplete IM features sulfomucins and it has been linked to the highest risk of developing GC [33–36]. As mentioned above, the progression through the first steps of the cascade depends on* H. pylori* virulence and environmental factors, as well as host genetic susceptibility. In particular, infection with cag-positive and/or vacA s1m1 strains is associated with more severe progression of the precancerous cascade [37, 38]. Independent epidemiological and animal studies have confirmed the sequential progression of these lesions [39, 40] and long-term follow-up studies estimated a risk assessment of 0.1% for AG, 0.25% for IM, and 0.6 to 6% in case of dysplasia for the development of gastric cancer [41, 42]. Despite having a well-characterized morphological basis for the precancerous cascade, studies showing the underlying molecular mechanism of progression through its steps are sparse. Shimizu et al. [43] have recently completed a comprehensive mutational evaluation of nontumorous gastric mucosa with* H. pylori* infection. The finding of low-abundance mutations in various genes in* H. pylori-*infected gastric tissue samples also supports the concept that other molecular mechanisms are driving the precancerous cascade of gastric cancer.

## 3. Molecular Bases of Gastric Carcinoma and Precancerous Cascade

Beyond the conventional classifications of gastric cancer, the heterogeneity of the disease has begun to be unraveled by a comprehensive mutational evaluation of primary tumors by several groups [10, 19]. The Cancer Genome Atlas (TCGA) network [10] proposes four molecular subtypes: (i) tumors positive for Epstein-Barr virus, characterized by recurrent PIK3CA mutations, extreme DNA hypermethylation, and amplification of JAK2, PD-L1, and PDCD1LG2; (ii) microsatellite-unstable tumors, which show elevated mutation rates, including mutations of genes encoding targetable oncogenic signaling proteins; (iii) gnomically stable tumours, enriched for diffuse-type carcinomas and for mutations of RHOA or fusions involving RHO-family GTPase-activating proteins; (iv) tumors with chromosomal instability, which present with marked aneuploidy and focal amplification of receptor tyrosine kinases [10]. More recently, Cristescu and coworkers [19] developed another molecular classification to provide novel translational relevant information. These molecular subtypes include (i) mesenchymal-like type, with the worst prognosis and tendency to occur at an earlier age; (ii) microsatellite-unstable tumors, which are hypermutated intestinal-subtype tumors occurring in the antrum with the best overall prognosis and the lowest frequency of recurrence; and (iii) the tumor protein 53- (TP53-) active and TP53-inactive types, including patients with intermediate prognosis and recurrence rates (TP53-inactive) and better prognosis (TP53-active) [19]. Although both classifications provided framework for further research, the finding of low-abundance mutations in multiples genes supports the concept that other mechanisms should participate in the molecular bases of gastric cancer. In this scenario, a striking finding in the TCGA network was the extreme DNA methylation in both promoter and nonpromoter CpG islands of the human genome. This was found across all molecular subtypes of gastric cancer, even more extensively than was previously studied in any tumor type [10]. These unexpected findings are the main focus of this review.

## 4. Host Genetic Factors: SNPs in Noncoding Regions

Recent data highlighted the role of SNPs in noncoding regulatory sites of inflammatory genes, detoxification enzymes, and/or protein-coding tumor suppressors and oncogenes in the pathogenesis of gastric cancer [17, 44, 45]. These polymorphisms also drive the progression of the precancerous cascade. As shown in Table 1, the SNPs at position −31 (T<C) of IL-1B result in the inhibition of gastric acid secretion and have been associated with noncardia and intestinal-type gastric cancer among Caucasians [46]. The presence of single A/T SNP at position −251 from the transcription start site (TSS) in the promoter region of the IL-8 gene has been associated with an increase in IL-8 production and linked with an increased risk for gastric cancer at cardia location [47]. Similarly, A/G polymorphisms at −1082 in IL-10 have been reported in association with intestinal-type of gastric cancer at cardia location [48]. Other inflammatory response gene polymorphisms have been linked to a rapid progression of the precancerous cascade, such as those associated with mediators of the innate immune response. An example is the 22-bp nucleotide deletion (−196 to −174 del) in the promoter region of the TLR-2 gene [49]. Among detoxification enzymes CYP2E, a member of the cytochrome P-450 superfamily, is a naturally ethanol-inducible enzyme involved in the metabolic activation of low molecular weight compounds such as N-nitrosamines [50]. It has been reported that polymorphisms −1295 C/G and −1055 C/T of CYP2E1 alter the transcriptional activity of the gene and can be related to an increased risk of gastric cancer in a synergic relation with other detoxification enzymes, such as the glutathione S-transferase (GST) [51] (Table 1). Among polymorphisms associated with protein-coding tumor suppressors and oncogenes, MET is a crucial gene to multiple oncogenic pathways and metastatic behavior. Polymorphisms in the promoter region of MET associated with the progression of gastric cancer have been reported [52, 53]. The most common substitution is −304 C>A, which can alter the junction sites for putative transcription factors such as Sp1 and AP-1/AP-2. The promoter region of the CDH-1 gene has been associated with DNA hypermethylation in the polymorphism −160 Increase risk of gastric cancer DNA methylation pattern of the promoter region of CDH1 [54] (Table 1). Taken together, SNPs of inflammatory response genes, detoxification enzymes, and/or protein-coding tumor suppressors and oncogenes modify the balance towards a more rapid progression of the gastric precancerous cascade. These SNPs may provide clues to explain the variation in gastric cancer risk across populations. As biomarkers, the prognostic role of SNPs can be easily determined from a blood sample to assess gastric cancer risk in individual patients.

## 5. DNA Methylation in Regulatory Regions of Protein-Coding Tumor Suppressors and Oncogenes

**Figure 2:**
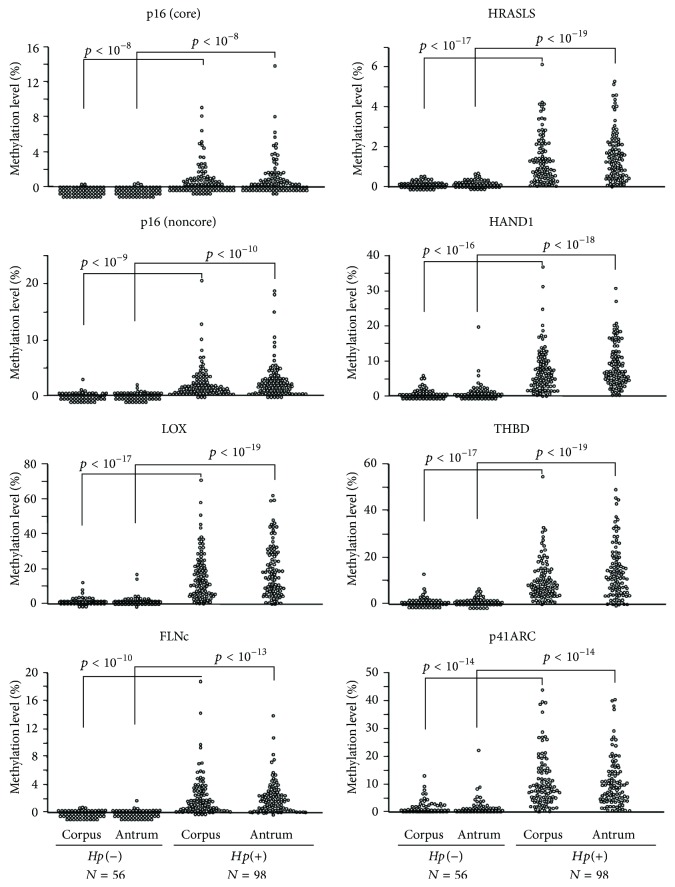
Higher levels of methylation are observed in gastric mucosae of* H. pylori*-positive volunteers than in* H. pylori*-negative volunteers. Methylation levels were measured in the corpus and antrum of 56* H. pylori*-negative volunteers and 98* H. pylori*-positive volunteers. All the eight CGIs (core region of p16, noncore regions of p16 and THBD; core regions of LOX, HRASLS, FLNc, and HAND1; and p41ARC exonic CGI) showed significantly elevated methylation levels (5.4- to 303-fold) in the* H. pylori*-positive volunteers. Methylation levels in the corpus were at the same levels as those in the antrum (taken with permission from [61]).

**Figure 3:**
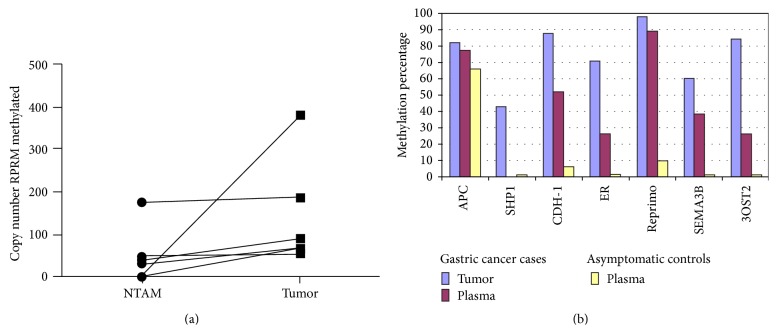
(a) RPRM methylation in tumor and nontumor adjacent mucosa (NTAM) tissues: higher methylation levels in tumor tissues are observed in comparison to NTAM in all six paired GC cases (taken from [63]). (b) Histogram representing the percentage of positive cases for Reprimo and other genes (APC, SHP1, CDH-1, ER, SEMA3B, and 3OST2) in 43 prospectively collected gastric cancer cases and 31 asymptomatic age- and gender-matched controls. Only Reprimo shows significant differences in plasma between gastric cancer and asymptomatic controls (taken with permission from [68]).

DNA methylation is an important event in the regulation of gene expression, affecting all of the pathways in the cellular network [55]. DNA methylation is a process in which cytosines acquire a methyl group in 5′-position only if they are followed by a guanine (CpG site) [56]. This modification results in gene silencing. In this scenario, an emerging catalog of gastric cancer genes altered by DNA methylation has been established [10]. However, there are limited reports addressing the role of DNA methylation in the gastric precancerous cascade. Two studies [57, 58] demonstrated that IM showed a higher methylation index than that of AG, but no differences between IM and dysplasia were observed. Specific genes, such as p16, display up to a 4-fold increase in their methylation status in the progression from dysplasia to gastric cancer. A study by Chan et al. [59] demonstrated that DNA methylation of the CDH-1 gene was associated with* H. pylori* infection (*p* = 0.002) [59]. This study, alongside another manuscript by Leung et al. [60] evaluated the presence of DNA methylation in the CDH-1 promoter region before and after eradication of* H. pylori*. Results from both studies suggest that* H. pylori* eradication therapy may reverse aberrant DNA methylation in patients with AG. A significant reduction in the methylation density of the promoter region of the CDH-1 gene was also observed [60]. Maekita et al. [61] expanded these findings to other genes in a healthy donor population. Methylation levels were 5.4- to 303-fold higher in* H. pylori*-positive than in* H. pylori*-negative subjects (*p* < 0.0001) (Figure 2). Schneider et al. [62] performed a quantitative analysis of the DNA methylation status of the promoter region of Reprimo (RPRM), a putative tumor suppressor gene in gastric cancer [63], which demonstrated an association of the methylation status of the gene with the presence of virulence factors in the infecting* H. pylori* strains. Taken together, these findings suggest that* H. pylori* infection potently induces aberrant DNA methylation. Moreover, DNA methylation not only is induced by* H. pylori* infection, but also can be reversed by eradication therapy. In other words, aberrant DNA methylation may be considered to explain how environmental factors increase the susceptibility for cancer development [64]. An emerging body of evidence suggests that DNA methylation may be used as novel and specific biomarkers in gastric cancer [65, 66] through the measurement of circulating DNA in serum (cell-free DNA (cfDNA)). In this scenario, we have discovered that RPRM displays differences in methylation levels between nontumor adjacent mucosa (NTAM) and cancer tissue samples (Figure 3(a)) [63]. Methylation status of this gene may be assessed in plasma samples (Figure 3(b)) [67, 68], offering the opportunity for noninvasive detection of gastric cancer. Data presented here suggest that DNA methylation is a good example of how noncoding genomics not only participate in the pathogenesis of gastric cancer but also are a promising family of biomarkers for gastric cancer risk prediction and prognostication. Further validation of candidate methylation markers in independent cohorts will be required to translate to clinical applications.

## 6. Noncoding RNAs

The molecular dogma in biology presents a unidirectional flow of genetic information from DNA, which is transcribed to RNA, which is translated to protein. With the passage of time, it was discovered that the transfer of genetic information is not unidirectional. The most important evidence of this is that 80% of the human genome is transcribed, but only 2 to 3% encode for proteins [69]. This antecedent is supported by the strong correlation between increased organism complexity and the increment in the proportion of the noncoding genome [70]. More recently, it has become clear that noncoding RNAs (ncRNAs) are of crucial importance for normal development and physiology, as well as the development of disease [71]. ncRNAs are literally defined as RNAs that do not code for proteins. This includes all RNAs, except messenger RNAs [72]. Traditional classes of ncRNAs are also included, such as ribosomal RNAs (rRNAs) and transfer RNAs (tRNAs) involved in mRNA translation, small nuclear RNAs (snRNAs) implicated in splicing, and small nucleolar RNAs (snoRNAs) involved in the modification of rRNAs. In addition, ncRNAs can also be divided into small (~20–200 nucleotides; nt) and long RNAs (200 nt to ~100 kilobases) [73] (Table 2).

## 7. Small Noncoding RNAs

**Figure 4:**
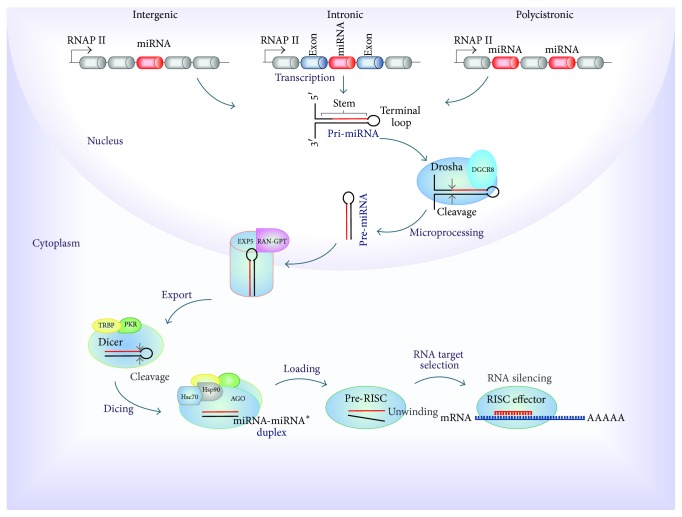
Canonical pathway of* miRNA *biogenesis in human. miRNAs are transcribed by* RNA polymerase II* (*RNAP II*) from* intergenic, intronic*, or* polycistronic* loci to long primary transcript, called* primary miRNA* (*pri-miRNA*), which consists in a stem, a terminal loop, and single-stranded RNA segments at both the 5′- and 3′-UTR sides.* Microprocessor complex* (*Drosha* and* DGCR8* cofactor) cleaves the stem-loop and releases a small hairpin-shaped RNA, called* precursor miRNA* (*pre-miRNA*). Following, pre-miRNA is exported into the cytoplasm by the transport complex formed by* protein exportin 5* (*EXP5*) and* GTP-binding nuclear protein RAN-GTP*. Subsequently, pre-miRNAs are cleaved by a ternary complex formed by* Dicer*,* TAR RNA Binding Protein* (*TRBP*), and* Protein Activator of PKR* (*PACT*), producing small RNA duplexes (*miRNA-miRNA* ^**∗**^). Next, these are loaded onto an* Argonaute protein* (*AGO*) to form an immature* RNA-Induced Silencing Complex* (*RISC*) or* pre-RISC*, in a process mediated for* Heat shock cognate 70-* (*Hsc70-*)* Heat shock protein* (*Hsp90*) chaperone complex. AGO protein separates the two strands to generate a mature* RISC effector*. Finally, RISC binds the target mRNA through complementary binding of 6 to 8 base pairs of the miRNA, with a specific sequence of the target resulting in the gene silencing.

Small ncRNAs (sncRNAs) are represented by PIWI-interacting RNAs (piRNAs), small interfering RNAs (siRNAs), and microRNAs (miRNAs). piRNAs are specialized for repression of mobile elements and other genetic elements in germ line cells (e.g., LINE1 piRNAs and piR-823) [74, 75]. piRNAs and their associated proteins PIWI have been reported as deregulated in various tumor types and associated with the carcinogenic process [71]. siRNAs regulate posttranscriptional gene silencing and the defense against pathogen nucleic acids (e.g., L1-specific siRNA and oocyte endo-siRNAs) [76, 77] (Table 2). Therefore, they seem to have great potential in disease treatment, especially in the silencing of oncogenes [73]. miRNAs were discovered in the decade of 90s by Lee et al. [78] that were studying the fetal development of* Caenorhabditis elegans*. The investigation revealed that lin-4 gene was responsible of control of various developmental phases of the nematode. Interestingly, instead of encoding for a protein, this gene was transcribed into a short string of noncoding RNA that regulated another gene called lin-14 [78]. To date, more than 30,000 miRNAs have been found in over 200 species [79]. In humans, the latest miRNA database miRBase release (v21, June 2014, http://www.mirbase.org/) contains 2,588 annotated mature miRNAs. It is estimated that 60% of coding genes may be regulated by miRNAs. MicroRNAs are defined as small (~22 nt) noncoding RNAs, highly conserved, involved in the posttranscriptional regulation of gene expression in multicellular organisms [80]. Most miRNAs are transcribed by RNA polymerase II (RNAP II) from intergenic, intronic, or polycistronic loci to long primary transcripts, called primary miRNAs (pri-miRNAs) [81]. A typical pri-miRNA consists of a stem of 33–35 bp, a terminal loop, and single-stranded RNA segments at both the 5′- and 3′-UTR sides. In the miRNA maturation process, two steps of cleavage take place. The first cleavage is performed in the nucleus by the Microprocessor complex (RNA III endonuclease, Drosha and DGCR8 cofactor) by cropping the stem-loop to release a small hairpin-shaped RNA of ~65 nucleotides in length, called precursor miRNA (pre-miRNA). Following Drosha processing, pre-miRNAs are exported into the cytoplasm by the protein exportin 5 (EXP5) forming a transport complex with GTP-binding nuclear protein RAN-GTP [82]. Upon export to the cytoplasm, pre-miRNAs are cleaved for the second time, by a ternary complex formed by RNase III endonuclease Dicer, TAR RNA Binding Protein (TRBP), and Protein Activator of PKR (PACT), producing small RNA duplexes about 22 nt in length [83]. The miRNA-miRNA^*∗*^ duplexes are made up by a “guide strand” and a “passenger strand” (miRNA^*∗*^). Selection of the guide strand depends on the relative thermodynamic stability of the first 1–4 bases at each end of the small RNA duplex [84]. Subsequently these are loaded onto an Argonaute protein (AGO 1–4) to form an immature RNA-induced Silencing Complex (RISC) or pre-RISC in a process ATP dependent mediated for Heat shock cognate 70- (Hsc70-) Heat shock protein (Hsp90) chaperone complex [85]. AGO proteins separate the two strands either via passenger-strand cleavage or through the aid of internal mismatches to generate a mature RISC effector [86]. Finally, activated RISC binds the target mRNA through complementary binding of 6 to 8 base pairs at the 5′ region of the miRNA guide strand (seed region), with a specific sequence of the 3′ region (3′UTR) of the target mRNA [87]. This affects both the stability and translation of messenger RNAs, resulting in downregulation of gene expression [20] (Figure 4). The biogenesis and function of miRNAs are under tight genetic and epigenetic control, including DNA methylation [88, 89]. Their deregulation is associated with many human diseases, particularly cancer [90, 91]. Oncogenic processes such as cell proliferation, differentiation, migration, and invasion are regulated by miRNAs [55]. This role has been associated not only with their binding to target genes, but also with the disruption of physiological expression patterns [91]. An example of this is the disruption of feedback loops between miRNAs and their target genes [92]. In gastric cancer cells, miR-139 could inhibit Jun expression by targeting a conserved site on its 3′-UTR, whereas Jun could induce miR-139 expression in a dose dependent manner through a distant upstream regulatory element which colocalizes spatially to miR-139 locus [93]. Functional analysis showed that restored expression of miR-139 significantly induces apoptosis and inhibits cell migration and proliferation as well as tumor growth through targeting Jun [93].

## 8. PIWI-Interacting RNAs in Gastric Cancer and the Gastric Precancerous Cascade

As shown in Table 3, specific sncRNAs expression patterns have been reported in gastric cancer, as is the case of the piRNAs [94]. Specifically, the expression level of piR-651 was significantly higher in gastric cancer tissues than in noncancerous tissue samples. Likewise, levels of this marker were low in early stages of gastric cancer (*p* = 0.032).* In vitro*, exogenous administration of piR-651 inhibitor in gastric cancer cell lines diminished proliferation rates and resulted in G2/M arrest [95]. In contrast, expression levels of piR-823 were significantly underexpressed in human gastric cancer tissue samples when compared with paired noncancerous tissues. It was further demonstrated that tumor growth was suppressed by piR-823, in* in vitro* and* in vivo* gastric cancer models [75]. Blood levels of piR-823 in gastric cancer patients were significantly lower than in those from healthy controls. In conjunction, piR-823 peripheral blood levels in patients with advanced tumor-node-metastasis stage and distant metastasis were found significantly lower than those in patients with early T stage and no metastasis. ROC curve analyses revealed that peripheral blood levels of piR-823 were a valuable biomarker for distinguishing gastric cancer from controls, with an area under the curve (AUC) of 0.822 (95% CI: 0.749–0.896, *p* < 0.0001), sensitivity of 81%, and specificity of 81% [96] (Table 3). piRNAs are short fragments and may pass through cell membranes, not be degraded, and be detected and isolated from body fluids [97]. This indicates that piRNAs have potential as molecular biomarkers in gastric cancer [98].

## 9. miRNAs in Gastric Cancer and the Precancerous Cascade

In gastric cancer, Ueda et al. [99] described 22 upregulated and 13 downregulated miRNAs, from paired tumor and nontumor adjacent mucosa samples. Intestinal- and diffuse-type gastric cancers showed different miRNA signatures. Eight miRNAs (miR-105, miR-100, miR-125b, miR-199a, miR-99a, miR-143, miR-145, and miR-133a) were upregulated in diffuse-type gastric cancer, and 4 miRNAs (miR-373^*∗*^, miR-498, miR-202^*∗*^, and miR-494) were upregulated in intestinal-type gastric cancer [99]. Analysis of miRNA expression patterns also revealed a progression in TNM stage according to higher expression levels of miR-125b, miR-199a, and miR-100 [100]. Low expression of let-7g and miR-433 and high expression of miR-214 were associated with poor overall survival, independent of depth of invasion, lymph-node metastasis, and TNM stage. Li et al. [100] identified miRNA expression profiles associated with differences in overall and relapse-free survival. In this study, let-7a, miR-126, and miR-30a-5p were protective and miR-10b, miR-21, miR-223, and miR-338 were associated with higher risk of relapse and poorer survival rates. Taken together, these findings show that miRNAs have differential expression in gastric cancer subtypes and are related to gastric cancer progression and prognosis.

The role of miRNAs in the gastric precancerous cascade has been addressed by Wang et al. [101]. Using an* in silico* approach from Gene Expression Omnibus (GEO) datasets, these authors identified 20 differentially expressed miRNAs from* H. pylori*-related AG and IM samples. These miRNAs were involved in pathways including signal transduction, cell growth and death, and transport and catabolism. Among the target genes, RAB22A, SOX4, IKZF2, PLAG1, and BTBD7 were simultaneously regulated by several differentially expressed miRNAs. On the other hand, miR-204 was decreased in* H. pylori*-related AG [102]. Knockdown of this miRNA promoted invasion and proliferation rates of gastric cancer cells* in vitro*. Downregulation of miR-204 and overexpression of SOX4 promoted the epithelial-mesenchymal transition process [102].

## 10. Potential Role of miRNAs as Noninvasive Biomarkers for Gastric Cancer

**Figure 5:**
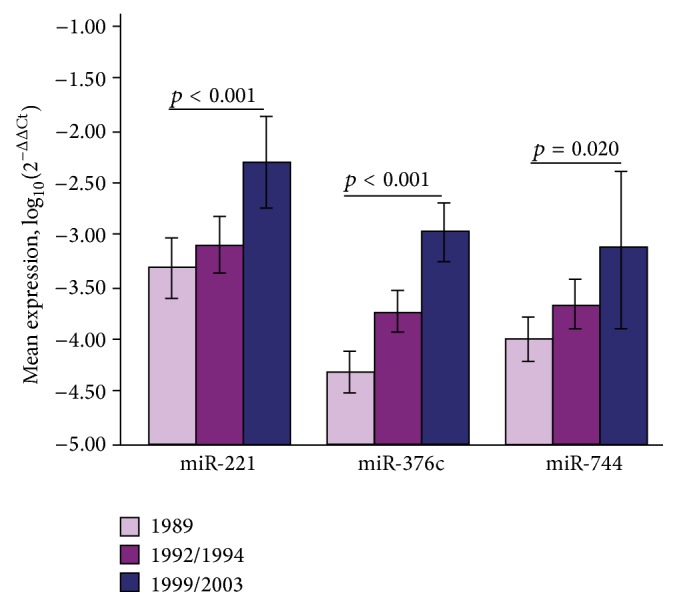
Marked increasing expression of miR-221, miR-376c, and miR-744 over time in 20 GC cases during 15-year (1989–2003) follow-up period. Error bars represent 95% CI (taken with permission from [103]).

Four studies examined the potential role of miRNAs as noninvasive biomarkers for gastric cancer in the context of the gastric precancerous cascade [103–106]. Song et al. [103] investigated the potentiality of serum miRNAs as biomarkers for early detection of gastric cancer in a population-based study in Linqu, a high-risk area of GC in China. Differential miRNAs were identified in serum pools of GC and control and validated gastric cancer and dysplasia versus controls pairs, respectively. The miRNA profiling results demonstrated that 16 miRNAs were markedly upregulated in gastric cancer patients compared to controls. Further validation identified a panel of three serum miRNAs (miR-221, miR-744, and miR-376c) as potential biomarkers for noninvasive detection of gastric cancer in a 15-year follow-up period (Figure 5). Receiver operating characteristic (ROC) curve-based risk assessment analysis revealed that this panel could distinguish gastric cancer with 82.4% sensitivity and 58.8% specificity. Fu et al. [104] detected the levels of circulating miR-222 in plasma of gastric cancer patients and evaluated its diagnostic value (Table 3). The result showed that the expression of circulating miR-222 in plasma was significantly upregulated in gastric cancer compared with AG and healthy controls (*p* < 0.001). ROC curve analyses revealed that miR-222 had a diagnostic accuracy of 0.850 with 66.1% sensitivity and 88.3% specificity. Liu et al., [105] examined the expression patterns of serum let-7 miR and its target gene, pepsinogen C (PGC) in gastric cancer, AG, and controls. The results showed that sera let-7c, let-7i, and let-7f demonstrated significant differences throughout the progression of the cascade (Table 4). The feasibility of using gastric juice was examined by Yu et al. [106]. Gastric cancer patients had significantly lower levels of gastric juice of miR-129-1-3p and miR-129-2-3p with an AUC of 0.639 and 0.651, respectively. Taken together, these results suggest that miRNAs play essential roles in gastric cancer at cell proliferation, cell cycle, and invasion/metastasis levels. In gastric precancerous conditions, miRNAs are linked with* H. pylori*-related gastritis and IM. MicroRNAs have strong potential as novel noninvasive biomarkers for gastric cancer risk assessment.

## 11. Long ncRNAs

Long ncRNAs (lncRNAs) make up the largest portion of the mammalian noncoding transcriptome [107]. They are essential regulatory molecules implicated in diverse cellular processes. Among them there are intergenic ncRNAs (lincRNAs), involved in chromatin remodeling and imprinting (e.g., HOTAIR and XIST) [108]; transcribed ultraconserved regions (T-UCRs) that regulate the expression of genes through interactions with miRNAs and hypermethylation of promoter CpG islands (e.g., uc.160 and uc.283A) [109]; and circular RNAs (circRNAs), which act as potent miRNA sponges and modulators of RNA-binding proteins (e.g., CDR1as-7 and SRY) [110] (Table 2). Recent reports suggest that aberrant expression of lncRNAs could play an important role in cancer [111]. Besides, the tissue specific nature of expression of lncRNAs makes them potentially advantageous for identification of biomarkers [112].

## 12. LncRNAs in Gastric Cancer and the Precancerous Cascade

In gastric cancer, HOTAIR overexpression has been correlated with advanced staging, lymph node metastasis, and poor overall survival [113, 114], suggesting it may serve as a biomarker for poor prognosis. A meta-analysis demonstrated that HOTAIR expression was a significant factor in the incidence of lymph node metastasis (present versus absent: OR 4.47, 95% CI: 1.88–10.63) and vessel invasion (positive versus negative: OR 2.88, 95% CI: 1.38–6.04) without heterogeneity across studies [115].* In vitro*, inhibition of HOTAIR in gastric cancer cells suppresses tumor invasion and reverses epithelial-to-mesenchymal transition [116]. Moreover, HOTAIR can act as a sponge for endogenous miR-331-3p, preventing the silencing of human epithelial growth factor receptor 2 (HER2) oncogene in gastric cancer [117]. Another lncRNA, LINC00152, revealed an increased expression level in gastric carcinoma in comparison with matched nontumor tissue and normal mucosa from healthy controls. In addition, high expression of LINC00152 was correlated with depth of invasion in gastric cancer patients. LINC00152 levels in gastric juice from patients with gastric cancer were also found significantly higher than those from controls [118]. Another study showed that the plasma levels of LINC00152 were significantly elevated in advanced and early stage gastric cancer patients when compared to healthy controls. Additionally, no differences were found in LINC00152 levels between plasma and exosomes [119], the latter being an important factor for protection of lncRNAs in plasma. These studies highlight the potential of LINC00152 as a noninvasive biomarker for the detection of gastric cancer. Another important lncRNA is H19. This is a maternally imprinted gene, which controls cell growth [97]. H19 has been reported as being upregulated in gastric cancer cells and gastric cancer tissue samples compared to controls [120]. Moreover, ectopic expression of H19 increased gastric cancer cell proliferation, inhibited cell apoptosis, and suppressed p53 activation [121]. Likewise, H19 can function as a primary microRNA precursor [122] and H19-derived miR-675 increases cell proliferation in gastric cancer cells by targeting the tumor suppressor RUNX1 [123]. At the same time, H19/miR-675 upregulation promotes proliferation, migration and invasion in gastric cancer cells, and tumorigenesis and metastasis in* in vivo* gastric cancer models [124]. A recent study showed that the expression levels of H19 in plasma were significantly higher in GC patients compared with controls. ROC curve analysis resulted in an AUC of 0.838, *p* < 0.001, with a sensitivity of 82.9% and specificity of 72.9%. Furthermore, plasma levels of H19 were significantly higher in dysplasia patients than in healthy controls, with an AUC of 0.877, sensitivity of 85.5%, and specificity of 80.1%. Plasma levels of H19 decreased in postoperative samples in comparison to preoperative samples. In addition, H19 expression showed high stability in the blood and no significant correlation with any type of blood cell in peripheral blood samples, which indicated that H19 expression in plasma may reflect tumor dynamics in GC patients. In conclusion, plasma H19 could serve as a potential biomarker for diagnosis of GC, in particular for early tumor screening [125] (Table 3).

In summary, data presented here suggests that molecular alterations beyond coding genes, that is, single nucleotide polymorphisms (SNPs), DNA methylation, miRNAs, and other noncoding RNAs, play a role in gastric cancer and the precancerous cascade. All these factors can ultimately be translated as novel and specific biomarkers which should impact gastric cancer mortality.

## Review Criteria

We searched PubMed for full text publications in English with no date limitations applied, although attention was centered on papers published in 2010–2015. A search was conducted under each of the main subsections of the paper. As such, search terms included a combination of gastric cancer, SNPs, ncRNA, miRNA, and DNA methylation, amongst others, to provide a comprehensive literature search. Additional papers were identified by crosschecking reference lists of previously identified papers. This review is representative of the topics discussed and, therefore, has not cited all reports.

## Figures and Tables

**Table 1:** Selected single nucleotide polymorphisms (SNP) in inflammatory response, detoxification, and cancer-related and DNA repair genes associated with increasing susceptibility of gastric cancer. The sign (−) indicates that the SNP is located in the direction of the promoter region, the sign (+) indicates that the SNP is located in the direction of the coding region, the number indicates the nucleotide position from TSS, and the first nucleotide is substituted (/) by the second (taken from [126]).

Gene	Variation allelic	SNP	Location	rs number	Effect	Reference
IL-1B	SNP	−511 T/C & −31 T/C	Near gene -5′	rs1143627	Increased IL-1 beta production and inhibition of gastric acid secretion	[46]

IL-8	SNP	−251 A/T	Near gene -5′	rs4073	Increased IL-8 production, associated with Lauren-class intestinal-type gastric cancer	[47]

IL-10	SNP	−1082 A/G	Intron region	rs1800896	Reported associated with the Lauren-class intestinal-type, cardia-located gastric cancer	[48]

TLR2	Deletion	−196 to −174del	Promoter region	Not applicable	Susceptibility to gastric cancer in the Southeastern Brazilian population	[49]

CYP2E1	SNP	−1295 C/G −1055 C/T	Near gene -5′ Near gene -5′	rs3813867 rs2031920	A risk factor for gastric cancer in Asians by a synergic relation with GSTM1	[51]

MET	SNP	−304 C/A	Near gene -5′	undefined	Activated the transcription at junction sites for putative transcription factors such as Sp1 and AP-1/AP-2	[52]

CDH1	SNP	−160 C/A	Near gene -5′	rs16260	Increased risk of gastric cancer DNA methylation pattern of the promoter region of CDH1	[54]

**Table 2:** Types of human noncoding RNAs.

Types	Subclasses	Symbol	Biological functions	Illustrative examples	References
Small noncoding RNAs (~20–200 nt)	PIWI-interacting RNAs	piRNAs	Repression of mobile elements and other genetic elements in germ line cells	LINE1 piRNAs; piR-823	[74, 75]
	Small interfering RNAs	siRNAs	Gene silencing and defense against pathogen nucleic acids	Oocyte endo-siRNAs; L1-specific siRNA	[76, 77]
	microRNAs	miRNAs	Posttranscriptional regulation of gene expression	lin-4; miR-139	[78, 93]

Long noncoding RNAs (200 nt to ~100 kb)	Intergenic noncoding RNAs	lincRNAs	Regulation of chromatin remodeling and imprinting	HOTAIR; XIST	[108]
	Transcribed ultraconserved regions	T-UCRs	Regulation of genes miRNAs and methylation	uc.160; uc.283A	[109]
	Circular RNAs	circRNAs	Modulation of miRNA and RNA-binding proteins	CDR1as; SRY	[110]

**Table 3:** Types of human noncoding RNAs in gastric cancer.

Types	Name	Expression	Putative biological processes involved	Biomarker potential	References
sncRNAs	piR-651	Upregulated (tissue)	Proliferation and cell cycle	Predictor of early diagnosis	[95]
	piR-823	Downregulated (tissue, cell, and peripheral blood)	Proliferation and cell metastasis	Noninvasive diagnosis biomarker	[75, 96]
	miR-222	Upregulated (plasma)	Tumor metastasis	Noninvasive biomarker for the early detection and prognosis	[104]

lncRNAs	HOTAIR	Upregulated (tissue)	Tumor metastasis and cell invasion	Predictor of lymph node metastasis and poor survival	[113, 115, 116]
	LINC00152	Upregulated (tissue, gastric juice, and plasma)	Cell invasion	Noninvasive diagnosis biomarker	[118, 119]
	H19	Upregulated (tissue, cell, and plasma)	Proliferation, tumorigenesis, migration, invasion, and cell metastasis	Noninvasive biomarker for early diagnosis	[98, 120, 124, 125]

**Table 4:** Expression of serum let-7c, let-7i, let-7f, and PGC in GC and precancerous lesions.

	*N*	CON	AG	GC	ANOVA *p*	AG versus CON *p*	GC versus CON *p*
lg let-7c	638	2.97 ± 0.83	2.79 ± 0.88	3.00 ± 0.80	0.017	0.028	0.667
lg let-7i	638	2.84 ± 0.79	2.77 ± 0.92	3.11 ± 0.71	<0.001	0.32	0.001
lg let-7f	638	2.39 ± 0.65	2.36 ± 0.66	2.56 ± 0.59	0.003	0.627	0.009
PGC	606	13.86 ± 14.56	14.32 ± 10.99	21.16 ± 27.17	0.002	0.027	0.001

CON: control; AG: atrophic gastritis; GC: gastric cancer; PGC: pepsinogen C.

Taken with permission from [105].
